# Genetic Identification of Potential Drug Targets for Dental Caries: A Mendelian Randomization Study

**DOI:** 10.1002/hsr2.72546

**Published:** 2026-05-18

**Authors:** Junlei Bi, Anqi Liu, Na Zhang, Yuxin Chen, Changqing Liu, Yongna Zhu, Hebao Wen, Caiyun Ma

**Affiliations:** ^1^ Anhui Engineering Research Center for Neural Regeneration Technology and Medical New Materials Bengbu Medical University Bengbu Anhui China; ^2^ State Key Laboratory of Molecular Medicine and Biological Diagnosis and Treatment (Ministry of Industry and Information Technology), School of Life Science Beijing Institute of Technology Beijing China; ^3^ College of Humanities and Health Bengbu Medical University Bengbu China

**Keywords:** dental caries, drug targets, genetics, Mendelian randomization, targeted therapies

## Abstract

**Background and Aims:**

Dental caries (DC), a chronic and multifactorial disease, affects billions of people globally, posing a significant public health challenge. Despite its prevalence, the molecular mechanisms underlying DC and effective pharmacological targets remain elusive. This study leverages Mendelian randomization (MR) and large‐scale genomic data to identify potential drug targets for DC.

**Methods:**

We utilized eQTL and genome‐wide association studies (GWAS) data to identify genes whose expression is causally linked to DC risk. The eQTL data were extracted from the eQTLGen consortium, and the GWAS data were obtained from the IEU OpenGWAS database. We performed MR analysis using the R package TwoSampleMR and validated the results in the FinnGen cohort. We integrated phenome‐wide association studies (PheWAS), gene ontology (GO), and protein–protein interaction (PPI) network construction to explore the potential roles of the identified genes. Molecular docking studies were conducted to identify potential therapeutic agents targeting TUBB.

**Results:**

We identified 32 genes whose expression is causally linked to DC risk. Further validation in the FinnGen cohort narrowed these down to nine key genes: EIF4G3, PREX1, ZFYVE19, TUBB, CBLL1‐AS1, EDAR, ADRA2A, BBS10, and TCTN1. These genes were analyzed to explore their roles in dental caries. Molecular docking studies identified paclitaxel and mebendazole as potential therapeutic agents targeting TUBB, with binding energies as low as −9.2 kcal/mol, indicating strong binding affinity.

**Conclusion:**

We identified nine novel candidate drug targets for DC through a comprehensive MR approach combined with multi‐omics analyses. These findings highlight the potential of these genes as therapeutic targets and provide valuable insights into the genetic basis of dental caries. The findings provide insights into the genetic basis of DC and highlight targets for future therapeutic development, though experimental validation is required.

## 1 Introduction

Dental caries, a chronic, progressive, multifactorial disease, is mainly due to the bacterial fermentation of carbohydrates, producing acidic by‐products that lead to localized destruction of the hard tissues of the dentition. It begins with microbial changes within the biofilm and is essentially a disruption of the balance between dental minerals and the oral biofilm [1]. In the early stages of caries, the tooth surface shows increased roughness or surface demineralization, followed by the gradual development of cavities. Continued progression of the disease leads to pulpal involvement and swelling, and eventually systemic signs and symptoms may appear [2]. As the global population continues to grow, so does the number of DC cases worldwide [3]. DC is one of the two major global oral diseases and has historically been recognized as one of the most important oral health burdens worldwide. Epidemiologically, the global prevalence of caries remains high, affecting nearly 2.4 billion permanent teeth and over 530 million children with primary teeth. The burden of dental caries remains with a person for their entire life, as humans typically have only two sets of teeth. Once the structure of a tooth is destroyed, the patient often requires lifelong restorations and daily maintenance. The high prevalence of DC not only affects the quality of life of patients but also imposes a heavy economic burden on society. Untreated permanent tooth decay remains the most prevalent disease among the 371 diseases and injuries assessed in GBD 2021, with 2.24 billion cases worldwide [4]. Although significant progress has been made in the study of DC over the years, its specific etiology, pathogenesis, and pharmacological targets at the molecular level have not been fully elucidated [5]. This remains a major research challenge for health professionals. In addition to existing preventive and curative measures, the development of new drug targets is essential to improve caries prevention and treatment strategies. Drugs with genetically based targets are more likely to be successful in clinical trials, so incorporating genetics into caries‐related drug development may be an effective strategy to accelerate this process [6].

MR is an epidemiological method that uses genetic variation as an instrumental variable to make causal inferences between risk factors and disease. The method is similar to a randomized controlled trial (RCT), and with the continued development of GWAS and other large‐scale genetic datasets, MR is an increasingly recognized and popular method [7, 8]. MR examines associations between genetic variants of single nucleotide polymorphisms associated with exposure levels as a means of exploring the effect of changing that risk factor on outcomes. Compared to observational studies, MR avoids the influence of confounding factors [9]. Because genetic variants are randomly assigned at the time of conception, MR analysis can provide insight into causality, which in turn can inform risk factors in clinical trials and associated drug targets [10].

In this study, we made several innovative findings in the field of dental caries research. First, we identified novel therapeutic targets for DC using an MR approach and large‐scale phenotype‐wide genomic association analysis data. This method combines rich data on genetic variation to infer causal relationships between exposure factors and disease outcomes, increasing the reliability of causal inference and controlling for confounding factors. Subsequently, nine potential drug targets for DC were validated through drug candidate prediction and molecular docking studies, and the binding ability and mechanism of action of these drug candidates to the targets were evaluated at the molecular level, which provided an important basis for optimizing and finalizing the drug candidates. In addition, we also performed gene enrichment analysis and protein interaction network construction, the combination of which can provide evidence at both the gene and protein levels to help identify potential therapeutic targets and provide more in‐depth biological background information to enhance the credibility of causal inference. The ability to validate these associations at the level of biological processes and molecular mechanisms can deepen our understanding of their mechanisms in caries genesis and treatment. Finally, we analyzed nine caries‐related drug target genes, which provide a reference for new caries therapeutic targets. By integrating MR, phenome‐wide association analysis, drug candidate prediction, enrichment analysis, and protein interaction network construction, caries can be better studied from a molecular point of view, which will provide more valuable and targeted information for caries drug therapy and an important basis for the subsequent development of prevention and treatment strategies. To our knowledge, this is the first MR study integrating multi‐omics and docking to propose potential druggable targets for dental caries.

## 2 Materials and Methods

### 2.1 Study Design

**FIGURE 1:**
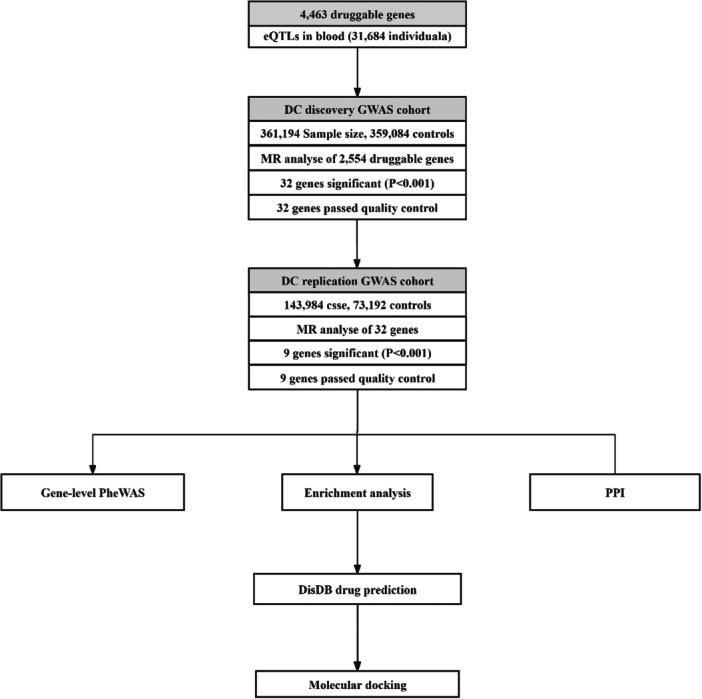
Overview of the study design. The figure illustrates the multi‐stage pipeline: (1) screening: two‐sample Mendelian randomization analysis was performed using blood eQTL data for druggable genes against the discovery GWAS cohort; (2) validation: significant genes were validated in an independent replication GWAS cohort; (3) characterization: validated genes underwent downstream analyses, including phenome‐wide association study, gene enrichment, and protein–protein interaction network construction; and (4) prediction: candidate drugs were predicted and evaluated via molecular docking.

This study employs a multi‐stage analytical strategy to systematically identify and validate potential drug target genes causally associated with dental caries risk. The overall research workflow is illustrated in Figure 1. Based on blood eQTL data, instrumental variables were selected from a pool of druggable genes. A two‐sample Mendelian randomization approach was then used to assess the causal association between gene expression and dental caries risk in the UK Biobank GWAS discovery cohort. Significantly associated genes were subsequently validated in an independent FinnGen GWAS cohort. Validated genes underwent further functional and phenotypic characterization, including polygenic effects and potential side effects via full‐phenotype association analysis, biological function exploration through GO and KEGG enrichment analysis, and network context interpretation via protein–protein interaction network construction. Finally, potential drug candidates were predicted using the DSigDB database, and their binding affinity and interaction patterns with corresponding target proteins were evaluated through molecular docking simulations, thereby forming a complete research loop from genetic causation inference to drug potential assessment.

### 2.2 Expose Resource

The eQTLs data were extracted from the eQTLGen consortium (https://eqtlgen.org/). This dataset comprises eQTLs from 31,684 predominantly healthy individuals of European ancestry, covering 16,987 genes [11]. To focus on potential drug targets, we restricted the analysis to SNPs located within ±5 kb of the transcriptional start or end sites of known druggable genes. This filtering yielded eQTLs for 2554 candidate druggable genes, which served as genetic instruments for gene expression levels in the Mendelian randomization analyses.

### 2.3 Outcome Data

The pooled GWAS data for DC used in this study (ukb‐d‐K02) were obtained from the IEU OpenGWAS (mrcieu.ac.uk) database of large‐scale GWAS, which contains 361,194 cases of European ancestry and 359,084 controls (total *N* = 720,278) of European ancestry. The validation set was obtained from FinnGen Release 10 (https://www.finngen.fi/en), released on December 18, 2023, which contains GWAS data for 143,984 DC replication cohorts and 73,192 controls (total *N* = 217,176).

### 2.4 Mendelian Randomization Analysis

In this study, we used the R package TwoSampleMR (version 0.5.6) to perform the MR analyses [12] and the built‐in function of the R package (harmonise_data) to import and harmonize the exposure and outcome data. In the selection of instrumental variables, we strictly adhered to the three main assumptions of MR: (1) the assumption of association: there is a strong correlation between SNPs and exposure factors; (2) the assumption of independence: SNPs are independent of confounders; and (3) the assumption of exclusivity: SNPs can only affect the outcome through the exposure factors [10]. During the course of the study, we first performed some quality control. In terms of population selection, the data in the training and validation sets consisted almost exclusively of people of European ancestry, which minimized potential selection bias to some extent. In addition, to exclude the effects of multiple validity and confounding factors, we further screened the data by instrumental variable sensitivity analysis, including gene multiplicity test and heterogeneity test. In selecting exposure SNPs, we used independent SNPs as instrumental variables and performed linkage equilibrium removal with the screening conditions of 10,000 kb and *r*² < 0.001 to minimize chained imbalances among instrumental variables, ensuring compliance with the core assumption of independence among instrumental variables in MR analysis. This stringent threshold was chosen as a sensitivity measure to minimize the risk of residual linkage disequilibrium influencing the causal estimates [12]. The Steiger filtering method was ultimately employed to exclude genes where the variation in SNP interpretation results exceeded the variation in exposure. In the MR analysis, the IVW method had the highest statistical power of all methods, provided that all genetic tools were valid [13]. Therefore, to follow the MR idea, we first performed a pleiotropy analysis with a *p*‐value of 0.05 as a condition to further ensure that there is no genetic pleiotropy between the exposure factors and the outcome and that the instrumental variables do not influence the outcome through factors other than the exposure factors. Subsequently, the instrumental variable (IVW) method was primarily employed to screen instrumental variables, yielding a total of 32 genes. To account for false positives from multiple testing, Bonferroni correction was applied to establish an adjusted significance threshold for multiple tests. In the discovery cohort only, a *p*‐value below 0.001 was defined as significant. These genes underwent iterative validation in the FinnGen cohort using a *p*‐value < 0.001 as the significance criterion, ultimately identifying nine significant genes. False discovery rate (FDR) correction was conducted by applied *q*‐value procedure, when FDR *q* < 0.1 was considered statistically significant, *p* < 0.05 but *q* ≧ 0.1 were considered to have a suggestive association [14]. In order to mitigate the impact of bias stemming from instrumental factors that are of a substandard quality, instrumental variables with an F statistic of less than 10 were eliminated. This calculation, denoted as F, is expressed as follows: F = (beta/se)² [15]. The range and mean F‐statistics for the instrumental variables used in the final analyses are provided in Supporting 7. The instrumental variables utilized demonstrated sufficient strength, with F‐statistics ranging from 29.709 to 14,552.869 and a mean F‐statistic of 301.562.

### 2.5 Phenome‑Wide Association Analysis

To further assess the horizontal pleiotropy and potential side effects of potential drug targets, we conducted a phenome‐wide association study (PheWAS) on the AstraZeneca PheWAS portal (https://azphewas.com/) [16]. The initial phase of the study used publicly available exome sequencing data from approximately 450,000 participants from the UK Biobank, covering approximately 15.5 thousand binary phenotypes and 1.5 thousand continuous phenotype data. Detailed construction methods can be found in the original article [16]. We performed multiple corrections and used a threshold of 2E‐5 as the default value in the AstraZeneca PheWAS portal to control for the risk of false‐positive results [17]. This threshold is established by the portal to regulate the error rate, thereby implementing a Bonferroni correction for the extensive number of phenotypes that are tested.

### 2.6 Enrichment Analysis

The power of enrichment analysis lies in its ability to assess the potential role of specific genes in the development of a disease or trait. Therefore, we performed gene ontology (GO) and Kyoto Encyclopedia of Genes and Genomes (KEGG) pathway enrichment analyses of the nine drug target genes using the R package clusterProfiler [18], Pathview [19], which covers biological processes (BP), molecular functions (MF), and cellular components (CC), and KEGG, which allows in‐depth analysis of the effects of genetic variation on complex biological processes, such as genetic variation and signaling pathways in organisms, to identify potential drug targets or drug discovery. To control for potential biases arising from pathway size and multiple testing, the enrichment analysis applied the Benjamini–Hochberg procedure for false discovery rate correction, which is the default and recommended adjustment method in the clusterProfiler R package used for this analysis. KEGG can deeply analyze the effects of genetic variations, signaling pathways, and other complex biological processes in organisms and discover potential drug targets or drug action mechanisms, providing new clues and ideas for the treatment of diseases.

### 2.7 Protein Interaction Network Construction

Key node proteins in the PPI network are often associated with multiple diseases and forms, and these proteins may be potential targets for disease treatment. Therefore, we generated PPI networks using the STRING database (https://string-db.org/) to explore potential interactions between nine drug target genes. The obtained results were then imported into Cytoscape for visualization. In addition, we performed PPI analysis using GeneMANIA (https://genemania.org/) [20].

### 2.8 Candidate Drug Prediction

The Drug Signatures Database (DSigDB) provides the link between drugs/compounds and their target genes. Currently, DSigDB contains more than 22,527 gene sets with 17,389 unique compounds covering 19,531 genes, providing researchers with extensive genetic data support. The main application of DSigDB is to perform gene set enrichment analysis, while DSigDB data can also be used to reveal the molecular mechanisms of diseases and provide new ideas and methods for disease diagnosis and treatment. Therefore, we used the DSigDB database (http://dsigdb.tanlab.org/DSigDBv1.0/) to further explore whether the identified drug target genes have the potential to become potentially effective interventional drugs by in‐depth study of the interactions between these proteins and drugs [21].

### 2.9 Molecular Docking

To evaluate the binding affinity and interaction patterns between candidate drugs and target proteins at the atomic level, molecular docking simulations were performed using AutoDock Vina 1.2.2 (http://autodock.scripps.edu/) [22]. The structural data of the drugs in question were obtained from the PubChem compound database [23] (https://pubchem.ncbi.nlm.nih.gov/). The corresponding identifiers are listed in Table 3. Protein structural data were obtained from the Protein Data Bank (PDB; http://www.rcsb.org/). The utilized PDB identifiers are enumerated in Table 3. Prior to the docking process, a series of preparatory steps was undertaken to ensure the optimal conditions for the experiment. This included the removal of water molecules and non‐essential ions from the protein structure, followed by the addition of polar hydrogen atoms. The docking grid box was centered on the known active site, with dimensions of 30 × 30 × 30 Å and a grid point spacing of 0.05 nm. The final docking results were then visualized using PyMOL.

## 3 Results

### 3.1 Thirty‐Two Genes Causally Significantly Associated With DC Risk During the Discovery Phase

**FIGURE 2:**
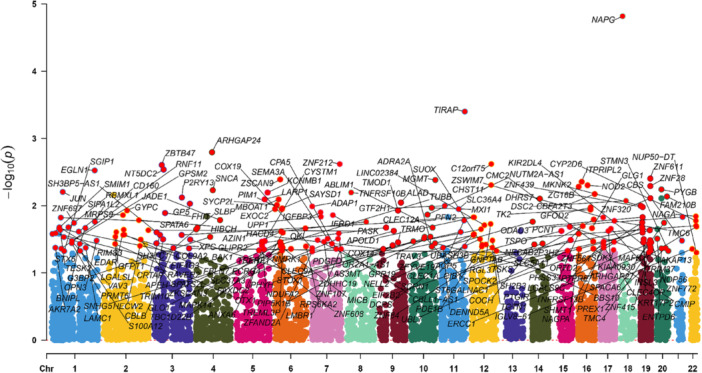
Manhattan plot of MR analysis in discovery phase. Each point represents a gene tested for a causal association with dental caries risk. The *x*‐axis shows the chromosomal position, and the *y*‐axis shows the strength of association from the inverse‐variance weighted MR method.

**FIGURE 3:**
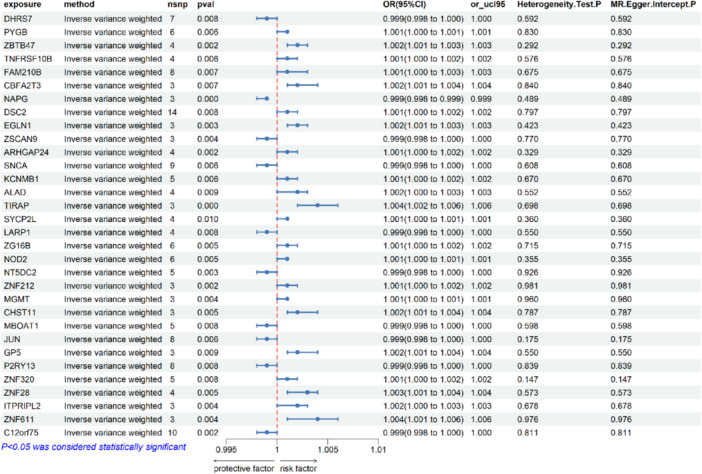
Forest plots displaying the findings from the discovery phase for 32 significant genes. Results are derived from the inverse variance weighted method. For each candidate gene significantly associated with dental caries risk, the plot shows the odds ratio with its 95% confidence interval, the corresponding *p*‐value, and the number of instrumental variable SNPs used in the analysis.

The ukb‐d‐K02 database contained a sample size of 361,194 cases and 359,084 controls of European origin, and by MR analysis, we obtained a causal relationship between the expression of 32 genes and the risk of dental caries from the initial set of 2554 druggable genes, and the specific results are shown in Figures 2 and 3. Specifically, using the IVW method, the initial estimation of the odds ratio (OR) for ZBTB47 was 1.002 (95% CI = 1.001–1.003, *p* = 0.002). Similarly, for CBFA2T3 in relation to the risk of DC was 1.002 (95% CI = 1.001–1.004, *p* = 0.007). The IVW method estimated an OR of 1.002 (95% CI = 1.001–1.003, *p* = 0.003) for EGLN1 in relation to the risk of DC. OR for TIRAP in relation to the risk of DC, using the IVW method, was 1.004 (95% CI = 1.002–1.006, *p* = 0.000). The IVW method estimated an OR of 1.002 (95% CI = 1.001–1.004, *p* = 0.005) for CHST11 in relation to the risk of DC. The estimated OR for GP5 in relation to the risk of DC was 1.002 (95% CI = 1.001–1.004, *p* = 0.009). OR for ZNF28 in relation to the risk of DC, using the IVW method, was 1.003 (95% CI = 1.001–1.004, *p* = 0.005). The IVW method estimated an OR of 1.004 (95% CI = 1.001–1.006, *p* = 0.004) for ZNF611 in relation to the risk of DC. During the analysis, we used a more stringent criterion, setting the *p*‐value at *p* < 0.001 and applying Bonferroni correction to 2554 drug targets. The final results of the three different analytical methods are listed in detail in the Supporting 1. The test data for pleiotropy and heterogeneity are fully documented in Supporting 2 and 3, respectively, for review and reference.

### 3.2 Replication Phase Nine Genes Remain Significant in Independent DC Cohorts

**FIGURE 4:**
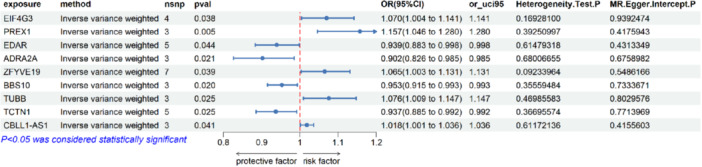
Forest plots displaying the findings from the replication phase for nine significant genes. The plot displays the causal associations of the nine validated candidate genes with dental caries risk. For each gene, the odds ratio with its 95% confidence interval is shown.

In the validation phase, we used GWAS data from the FinnGen database, which includes 143,984 cases and 73,192 controls of Finnish origin. In the analysis, we used the same method as in the training set, and the *p*‐value of 0.001 results showed a causal relationship between the genetic expression of nine genes and dental caries, as shown in Figure 4. An exhaustive record of the results obtained by the three analysis methods can be found in Supporting 4. In addition, the respective results of the pleiotropy and heterogeneity tests were organized and presented in Supporting 5 and 6, respectively.

### 3.3 PheWAS

To further explore the potential positive or negative effects of the nine identified potential drug target genes on other traits and to test whether potential pleiotropic effects were missed by the MR‐Egger intercept test, the present study adopted 17,361 dichotomous phenotypic data contained in the AstraZeneca PheWAS Portal database [16] along with 1419 quantitative phenotypic data and performed a phenotype‐wide genomic association study (PheWAS) at the gene level. The analysis was designed to reveal associations between the expression of proteins encoded by genes and specific diseases or physiological traits. The results are shown in Table 1 (CBLL1‐AS1 is not yet published on the website for further study, so the results are not shown), in which PREX1, BBS10, and TCTN1 drug targets were significantly correlated with other traits at the gene level (genomic correlation *P* < 5E‐5) [17], suggesting that the potential side effects of other drugs acting on these targets and the presence of horizontal pleiotropy in these genes may affect these targets. The remaining results are shown in Supporting Information Figures S1–S16.

**TABLE 1:** Traits significantly associated with PREX1, BBS10, and TCTN1 using AstraZeneca PheWAS portal.

Gene	ICD codes	Phenotype	Collapsing model	*p* value	No. samples
EIF4G3	Union#K593	Megacolon not elsewhere classified	ptv5pcnt	7.780E‐05	165,856
PREX1	131443	Source of the report of J11 (influenza virus not identified)	rec	2.080E‐05	424,049
EDAR	131837	Source of report of L98 (other disorders of skin and subcutaneous tissue not elsewhere)	flexdmg	5.360E‐05	443,874
ADRA2A	Union#D171	Benign lipomatous neoplasm of skin and subcutaneous tissue of trunk	raredmgmtr	5.250E‐05	6277
ZFYVE19	Union#K137	Other and unspecified lesions of oral mucosa	flexnonsynmtr	7.190E‐05	168,617
BBS10	41202#R31	Unspecified hematuria	ptv5pcnt	8.880E‐06	239,265
TUBB	40011#8243	Goblet cell carcinoid (mucocarcinoid tumor)	ptv	2.160E‐04	459,449
TCTN1	41202#J38	Diseases of vocal cords and larynx not elsewhere classified	raredmg	9.530E‐06	341,550

### 3.4 Enrichment Analysis

**FIGURE 5:**
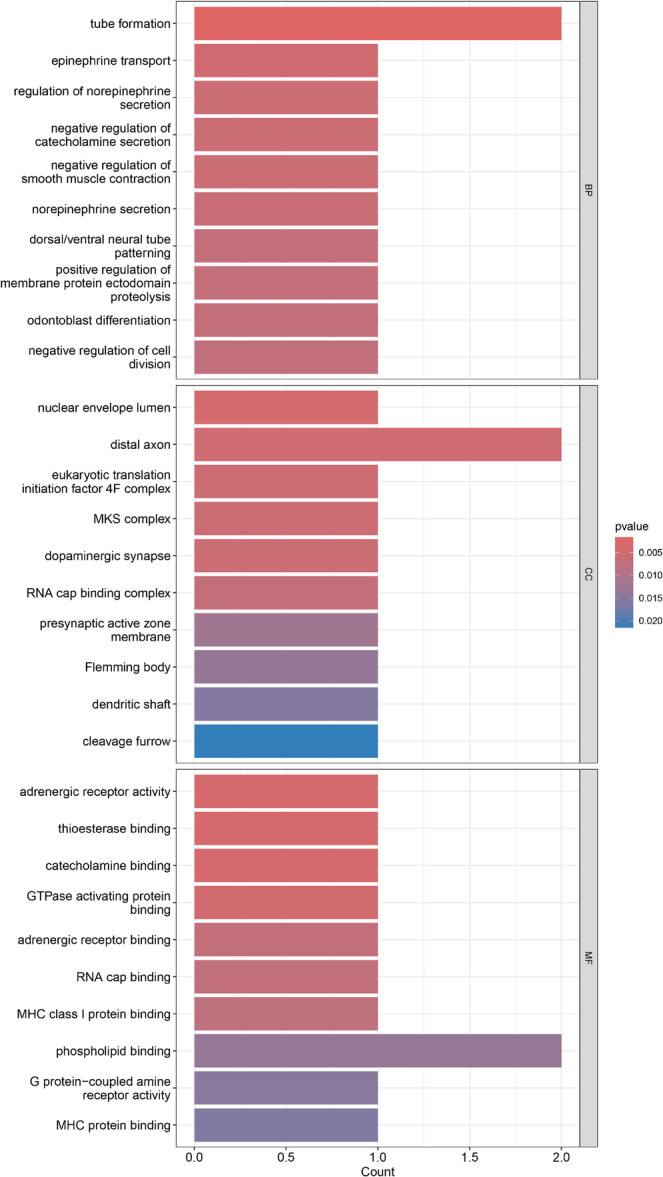
GO enrichment results for three terms. The bar chart shows the significant GO terms across biological process, cellular component, and molecular function categories.

**FIGURE 6:**
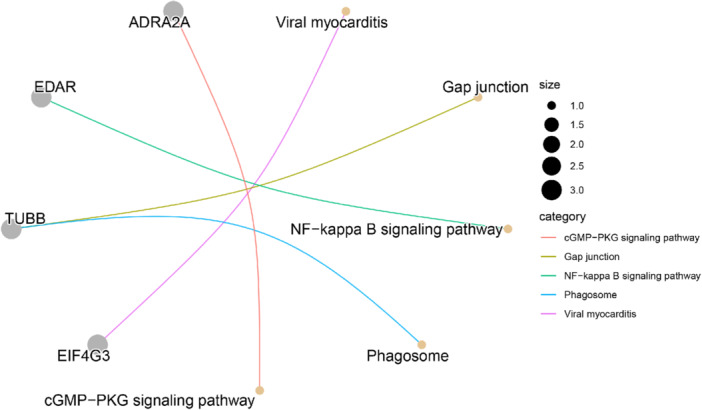
KEGG enrichment results. KEGG analysis was performed to identify biological pathways significantly associated with the validated candidate genes.

GO enrichment analysis is to find a class of overexpressed genes or proteins in a group of genes or proteins. It is usually a high‐throughput experiment, and the common ones are GO function annotation and KEGG pathway enrichment analysis. Through gene pathway enrichment analysis, we can first analyze the biological processes or signaling pathways that the genes may be involved in. As shown in Figure 5, the specific results of GO analysis have been clearly presented, covering the three main aspects of Biological Process (BP), Cellular Component (CC), and Molecular Function (MF). In addition, Figure 6 shows the detailed results of the KEGG analysis.

### 3.5 PPI Networks

**FIGURE 7:**
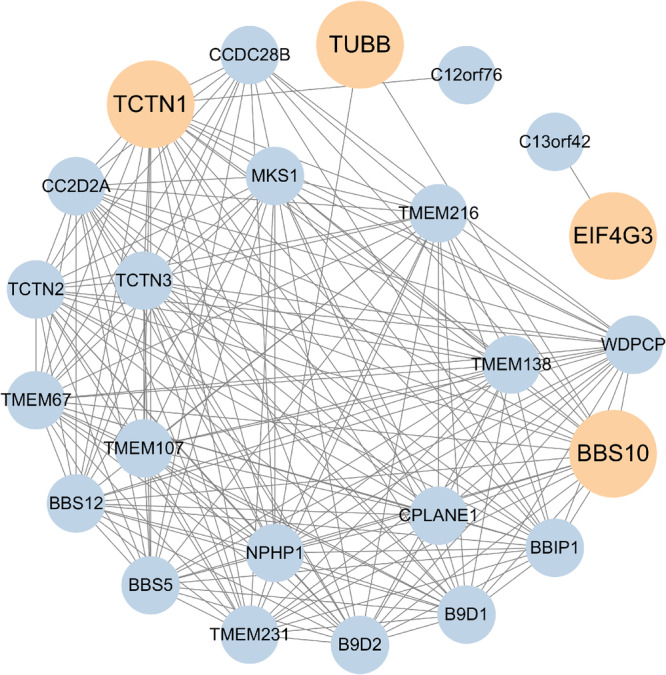
PPI network built with STRING. A PPI network was constructed to explore the functional associations and biological context of the nine validated candidate genes.

We entered nine drug target genes into the STRING database (https://cn.string-db.org/) to construct their interaction networks. We then visualized the generated data using Cytoscape software, and the obtained results are shown in Figure 7.

**FIGURE 8:**
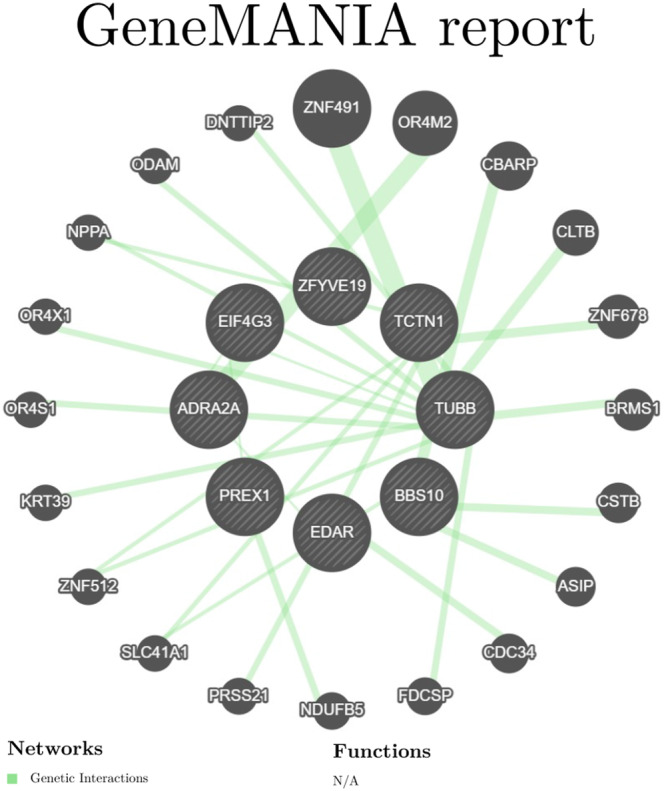
PPI network built with GeneMANIA. The network was generated to explore a wider functional context, showing predicted associations, including physical interactions, co‐expression, and pathway co‐membership, between the core candidate genes and related genes.

As shown in Figure 8, in the protein–protein interaction (PPI) network constructed using the GeneMANIA platform (URL: https://genemania.org/), in addition to the nine explicitly designated drug targets, the network covers an additional 19 potential, possibly interacting genes. These genes represent an important part of the network in addition to the known drug targets, further enriching the complexity and potential biological significance of the network structure.

### 3.6 Candidate Drug Prediction

In this study, the DSigDB database was used to predict potentially effective interventional drugs. We listed the candidate potential mixtures that were ranked in the top 10 based on the magnitude of the *p*‐value in Table 2. The results show that parthenolide (parthenolide MCF7 DOWN) is the most significant drug with which it interacts, while coumestrol (COUMESTROL CTD 00005717) interacts with most of the genes.

**TABLE 2:** Candidate drug predicted using DSigDB.

Drug names	*p* value	Enrichment odds ratio	Genes
Parthenolide MCF7 DOWN	0.002	35.413	BBS10;TUBB
BP 897 TTD 00002536	0.005	249.763	ADRA2A
Paclitaxel TTD 00010012	0.005	249.763	TUBB
Mebendazole TTD 00009145	0.005	249.763	TUBB
Brimonidine CTD 00000810	0.005	249.763	ADRA2A
COUMESTROL CTD 00005717	0.006	8.046	PREX1;TUBB;EIF4G3;ADRA2A
Dopamine TTD 00007706	0.006	208.115	ADRA2A
Oxymetazoline CTD 00006455	0.006	192.096	ADRA2A
Yohimbine CTD 00007005	0.008	146.868	ADRA2A
Phentolamine CTD 00006516	0.008	146.868	ADRA2A

*Note:* Enrichment odds ratios shown are derived from the DSigDB gene set enrichment analysis.

### 3.7 Molecular Docking

**FIGURE 9:**
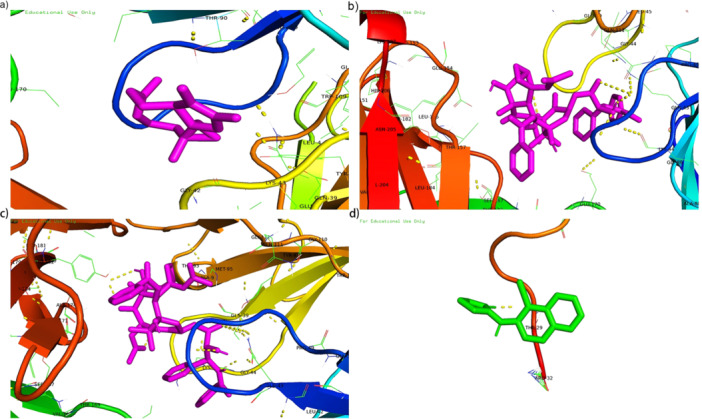
Docking results of available proteins small molecules. (a) TUBB docking Parthenolide. (b) TUBB docking Paclitaxel. (c) TUBB docking Mebendazole. (d) ADRA2A docking Brimonidine. The close‐up view shows the predicted binding conformation and specific interactions of a candidate drug within the binding pocket of its target protein, illustrating the structural basis for the calculated binding affinity.

In order to evaluate in depth the binding strength, that is, affinity, of the drug candidates to their targets and to more accurately reveal the druggable potential of the drug targets, this study was specifically analyzed in detail using molecular docking techniques. During the analysis of this study, we used Autodock Vina v.1.5.7 to obtain the binding sites and interactions of the first five drug candidates with the proteins encoded by the corresponding genes and to generate the binding energies for each interaction, yielding effective docking results for a total of four proteins with the drugs (Table 3 and Figure 9). Each drug candidate was bound to the protein target by visible hydrogen bonding and strong electrostatic interactions. The lowest binding energy of TUBB and mebendazole was −9.2 kcal/mol, indicating a very stable binding energy.

**TABLE 3:** Docking results of available proteins with small molecules.

Target	PDB ID	Drug	PubChem ID	Binding energy
TUBB	3QNZ	Parthenolide	7251185	−7.0
TUBB	3QNZ	Paclitaxel	36314	−9.1
TUBB	3QNZ	Mebendazole	4030	−9.2
ADRA2A	P08913	Brimonidine	2345	−7.4

## 4 Discussion

In this study, a combination of multiple MR methods and in‐depth mining of a large amount of genetic data identified nine genes whose genetic expression was causally associated with dental caries, namely EIF4G3, PREX1, EDAR, ADRA2A, ZFYVE19, BBS10, TUBB, TCTN1, and CBLL1‐AS1, which strengthened the causal inference reliability and controlled confounders. Meanwhile, this study systematically verified the underlying biological mechanisms by multi‐omics analysis (PheWAS, GO/KEGG, and PPI network). The medicinal value of these targets was further validated using drug candidate prediction and molecular docking techniques. It was shown that drugs such as Paclitaxel and Mebendazole had a high affinity (minimum binding energy of −9.2 kcal/mol) for the target protein (TUBB), indicating that the binding energy was very stable, suggesting that they could be used as potential drugs for caries treatment.

EIF4G3 is an important component of the eukaryotic translation initiation 4 F (EIF4F) complex, which is involved in processes such as mRNA cap recognition and mRNA transport to the ribosome, and its aberrant function may affect the synthesis of tooth‐associated proteins, which in turn may affect normal tooth development, with a potential link to the development of DC [24]. PRX1 expression is associated with tooth morphogenesis via Wnt5a, which affects molar morphology and mesenchymal stem cells (MSCs) proliferation, and it plays an important role in tooth development as a marker gene for MSCs. Mutations in the Prx1 gene cause inhibition of molar development and hypoplasia, which not only affects tooth function but can also result in poorly aligned teeth and an increased risk of dental caries [25]. EDAR is a member of the TNF receptor superfamily that binds to the heterodimer EDA‐A1, thereby activating the NF‐κB signaling pathway, which regulates the expression of proteins involved in enamel formation [26]. ADRA2A encodes the α2‐adrenergic receptor, whereas stimulation of the α2‐adrenergic receptor inhibits salivary secretion and blockade increases salivary secretion, suggesting that the receptor encoded by ADRA2A is involved in the process of regulating salivary secretion. It influences the regulation of the salivary glands by affecting the autonomic nervous system, which in turn affects the amount of saliva secreted [27]. Reduced salivary secretion rates have been reported to increase susceptibility to DC [28]. ZFYVE19 has been studied primarily in the context of liver disease and ciliary function [29], and the present study found that its genetic expression is associated with DC and may be a potential drug target. The protein encoded by BBS10 is involved in the formation of molecular chaperone complexes and assists in the assembly of the BBSome complex [30], which is one of the genes associated with Bardet–Biedl syndrome (BBS), and studies of the molecular mechanisms of the disease have provided direction for drug target research [31]. TUBB, an isoform of β‐microtubulin that assembles with α‐microtubulin to form microtubules, is widely distributed in cellular processes and may have potential effects on the function and development of tooth‐associated cells [32]. At the stage of cell differentiation, TUBB proteins are important for the differentiation of enamel‐forming and dentin‐forming cells. TCTN1 was found to be a potential therapeutic target for oral squamous cell carcinoma, and the knockdown of TCTN1 inhibited OSCC cell proliferation, migration, and invasion [33]. Its potential as a drug target has also been demonstrated in caries research. Meanwhile, another study explored the molecular mechanisms and potential therapeutic strategies for cilia‐related diseases, laying the groundwork for drug development against the cilia protein (TCTN1) [34]. Based on the properties of lncRNAs, the development of drugs targeting lncRNAs is a current research hotspot [35]. These results suggest that the drug targets proposed in this study have some relevance and development prospects for dental caries and have high research value.

Conventional caries treatment usually requires the removal of a large amount of tooth tissue, whereas medication is a non‐invasive treatment that is more cost‐effective and desirable in the long run [36, 37]. Paclitaxel is one of the most successful and widely used anticancer drugs [38]. Bacteria is the key factor of the four factors of caries etiology [36], theoretically, paclitaxel can also affect the normal cell cycle of caries‐causing bacteria, inhibit the normal growth of caries‐causing bacteria, and prevent the further development of DC. Some studies have shown that paclitaxel has the ability to regulate immune cells, enhance the body's clearance of caries‐causing bacteria, and indirectly inhibit the function of DC. Paclitaxel is one of the most successful and widely used anticancer drugs due to its unique anticancer mechanism, which induces apoptosis of cancer cells by promoting microtubule protein assembly and inhibiting microtubule depolymerization to prevent mitosis [39]. Although chamomile lactone is mainly used as an anti‐cancer drug, it also has good anti‐inflammatory effects [40]. Studies have confirmed that chamomile lactone can inhibit cytokines such as IL‐6 and TNF‐α and block their signaling, effectively reducing the inflammatory response [41]. And caries trigger the oral immune response by inducing the production of cytokines such as IL‐6, IL‐1, IL‐8, and TNF‐α. Therefore, chamomile lactone may reduce the inflammatory response and protect teeth from inflammatory damage. In addition, chamomile lactone may also regulate the function of immune cells and alter the body's immune defenses against caries‐causing bacteria. Studies have shown that chamomile lactone can inhibit the production of IL‐4 mRNA and IL‐4 protein in activated peripheral blood T cells [42]. Mebendazole is mainly used as a broad‐spectrum anthelmintic, and its biological mechanism is also mainly to inhibit the polymerization of microtubule proteins, which interferes with the normal physiological function of cells [43]. The association between mebendazole and DC requires further studies to clarify its role. Brimonidine is a selective α2‐adrenergic receptor agonist that acts primarily by activating ADRA2A receptors [44]. ADRA2A receptors are expressed in the salivary glands and are involved in the regulation of salivary secretion [27]. Brimonidine may affect salivary secretion by activating ADRA2A receptors, thereby affecting the oral environment and reducing the risk of DC. It is particularly important to emphasize that all computational predictions, including molecular docking results, represent preliminary computer simulation evidence. The primary value of this research currently lies in proposing hypotheses, establishing priorities, and guiding future research directions—it cannot directly prove clinical efficacy. The journey from identifying a target to successfully developing a therapy requires multiple steps, including validation of the target's biological function, lead compound screening and optimization, and in vitro and in vivo pharmacodynamic and toxicological evaluations. Currently, we are conducting relevant animal experiments to better support the findings of this study.

Our study has several strengths. First, our MR findings extend the insights from previous large‐scale GWAS on dental caries [45, 46]. While those studies successfully identified numerous genetic loci associated with caries susceptibility, our analysis moves beyond association to infer causality by integrating gene expression data. At the same time, we integrated MR and PheWAS for the first time, which significantly improved the reliability of causal inference while allowing a comprehensive assessment of drug target pleiotropy and potential side effects. Through the two‐step validation approach, after the causal association is identified by MR, PheWAS can further verify whether the association is specific or not and exclude false positives due to pleiotropy, further enhancing the robustness of the results. In addition, through PPI and molecular docking studies, biological processes are analyzed more comprehensively and deeply at the molecular level, and a closed‐loop validation from genes to drug targets is formed, which provides a new way of thinking for DC drug development. Enrichment analysis deeply mines biological information, which can deeply understand the mechanism of DC, analyze the mechanism pathways related to DC, and develop relevant drugs targeting these keys signaling pathways, providing new drug choices for the treatment of DC. The prediction of drug candidates accurately reveals the druggable value of these drug targets, and the study shows that the binding energy is very stable, which provides a potential drug direction for caries drug therapy and promotes the process of gene research to actual drug application. More importantly, our approach revealed novel candidate targets (TUBB and BBS10) not highlighted in prior GWAS, showcasing the unique value of MR in uncovering potential drug‐gable mechanisms.

Our study has several limitations. Although Mendelian randomization can verify causal relationships, it lacks some in vitro or in vivo experiments to support its actual efficacy. Also, MR methods have some limitations, such as genetic variants with pleiotropic effects that are successfully associated with outcomes may not be valid genetic tools. Multiplicity is the phenomenon that a gene or genetic variant can affect more than one phenotypic trait and can be a source of bias in MR analysis [10, 47]. In addition, our study data are primarily from populations of European ancestry, with limited diversity in the study cohort and issues of sample homogeneity, which may limit the generalizability of the results to other ethnic groups [48]. Mendelian randomization studies rely heavily on publicly available databases. There may be bias in the quality or coverage of the study data, affecting the accuracy of the results. Enrichment analysis, although valuable for functional exploration of the nine drug target genes, has limitations. As multiple tools or methods were not used for cross‐validation, the analysis results of a single method may be imprecise, and the results should be interpreted with caution. In terms of predicting drug candidates, the study was based on the DSigDB database for initial screening, which did not fully take into account the actual situation of drugs in vivo. Regarding molecular docking, while the calculated binding energies suggest favorable interactions, no re‐docking or RMSD calculations were performed to validate the docking protocol reliability, representing a computational limitation of the current study. And molecular docking studies only verified the binding of some drugs to their targets, and although potential drug targets can be identified, their efficacy in the clinical setting cannot be guaranteed. Therefore, this study is exploratory in nature to a certain extent. Simultaneously, this study serves as a comprehensive hypothesis‐generating effort. The transition from these computational targets and drug candidates to clinical applications requires an extensive sequence of experimental validation, including mechanistic studies in disease‐relevant models, medicinal chemistry optimization, and rigorous clinical trials.

## 5 Suggestions for Future Research

As there are still some limitations in the study, I would like to make some suggestions for future studies. To minimize bias and improve the generalizability of the study results across different races, subsequent studies could repeat the MR analysis in different racial populations, such as Asian and African populations, to verify the generalizability of the target genes. Multi‐omics integration studies can be performed. For example, more detailed data from other biological fields, such as proteomics, metabolomics, and transcriptomics, can be integrated into the study of dental caries to comprehensively understand the interactions and regulatory networks of genes, proteins, and metabolites in the process of caries development, to deeply analyze the complex biological mechanisms involved, and to improve the accuracy and reliability of the discovery of biomarkers and drug targets for dental caries. Any measurement error may affect the validity of Mendelian randomization estimation results and weaken the observed associations, and we should be cautious and need to further validate them with more accurate measurement techniques and larger data sets and assess the medical value of these targets through clinical trials to evaluate their safety and efficacy. The above recommendations will be taken into account in future studies to effectively translate the findings into clinical applications and develop new therapeutic approaches and strategies, with the ultimate goal of reducing the global caries burden and improving oral health.

## 6 Conclusion

In summary, our study successfully identified nine novel genetic targets (EIF4G3, PREX1, EDAR, ADRA2A, ZFYVE19, BBS10, TUBB, TCTN1, and CBLL1‐AS1) for dental caries through a comprehensive MR approach combined with multi‐omics analyses, including phenome‐wide association studies, enrichment analyses, protein–protein interaction network construction, and molecular docking. These findings highlight the potential of these genes as therapeutic targets and provide valuable insights into the genetic basis of DC. The identified drug candidates, such as paclitaxel and mebendazole, demonstrate promising binding affinity to their targets, suggesting their potential for future DC treatment. Overall, our research offers a strong foundation for further exploration of targeted therapies and advances the understanding of the molecular mechanisms underlying DC.

## Author Contributions

**Junlei Bi:** conceptualization, validation, writing – original draft, writing – review and editing. **Anqi Liu:** conceptualization. **Na Zhang:** methodology, software, data curation. **Yuxin Chen:** methodology. **Changqing Liu:** formal analysis, resources, project administration. **Yongna Zhu:** validation, supervision. **Hebao Wen:** validation, visualization. **Caiyun Ma:** investigation, supervision, funding acquisition.

## Disclosure

The lead authors Hebao Wen and Caiyun Ma affirm that this manuscript is an honest, accurate, and transparent account of the study being reported; that no important aspects of the study have been omitted; and that any discrepancies from the study as planned (and, if relevant, registered) have been explained.

## Ethics Statement

The data used in this study have been ethically approved by the Ethics Committee of Bengbu Medical University (Bengbu, China; approval no. 2024‐147).

## Conflicts of Interest

The authors declare no conflicts of interest.

## Supporting information


**Figure S1:** Binary traits PheWAS association with EIF4G3.

**Figure S2:** Continuous traits PheWAS association with EIF4G3.

**Figure S3:** Binary traits PheWAS association with PREX1.

**Figure S4:** Continuous traits PheWAS association with PREX1.

**Figure S5:** Binary traits PheWAS association with EDAR.

**Figure S6:** cContinuous traits PheWAS association with EDAR.

**Figure S7:** Binary traits PheWAS association with ADRA2A.

**Figure S8:** Continuous traits PheWAS association with ADRA2A.

**Figure S9:** Binary traits PheWAS association with ZFYVE19.

**Figure S10:** Continuous traits PheWAS association with ZFYVE19.

**Figure S11:** Binary traits PheWAS association with BBS10.

**Figure S12:** Continuous traits PheWAS association with BBS10.

**Figure S13:** Binary traits PheWAS association with TUBB.

**Figure S14:** Continuous traits PheWAS association with TUBB.

**Figure S15:** Binary traits PheWAS association with TCTN1.

**Figure S16:** Continuous traits PheWAS association with TCTN1.

**Table S1:** Results of the three Mendelian randomization methods in the discovery phase.

**Table S2:** Results of MR‐Egger intercept horizontal pleiotropy test at the discovery stage level.

**Table S3:** Results of heterogeneity test at the discovery phase.

**Table S4:** Results of the three Mendelian randomization methods in the replication phase.

**Table S5:** Results of MR‐Egger intercept horizontal pleiotropy test at the replication stage level.

**Table S6:** Results of heterogeneity test at the replication phase.

**Table S7:** Results of the F‐statistics for the instrumental variables.

## Data Availability

The data that support the findings of this study are available in the Supplementary Material of this article.
